# Characterization and expression analysis of *UBC* gene family provide insights into the potential roles in pigment biosynthesis in pepper fruit

**DOI:** 10.1186/s12864-026-12536-x

**Published:** 2026-06-09

**Authors:** Liqiang Jia, Zhiying Gou, YuXiang Wu, XiuWan Yang, Ding Lin, Wenzixi He, Di Wu, Laixue Pan, Maolian Wang, Jianwen He

**Affiliations:** 1 School of Biological Sciences, GuiZhou Education University, Guiyang, 550018 China; 2 Chili Pepper Research Institute, Guizhou Academy of Agricultural Sciences, Guiyang, 550002 China

## Abstract

**Background:**

Ubiquitin-conjugating enzymes (UBCs) are a critical part of the ubiquitin-proteasome pathway and play diverse roles in growth, development, and abiotic stress responses in pepper (*Capsicum annuum L.*). Although *UBC* genes have been reported in several plant species, genome-wide characterization of this gene family remains unexplored in pepper.

**Results:**

In this study, 56 putative CaUBCs were identified in pepper, which were clustered into 11 groups based on phylogenetic analysis, including ubiquitin E2 enzyme variant (UEV), SOMO (HUS), and COP10. CaUBCs within each subgroup showed relatively conserved domains and motifs. Most *CaUBC* genes contain plenty of light or stress response elements. qRT-PCR analysis demonstrated distinct expression patterns of *CaUBCs* in the pericarp and placenta during fruit development, as well as in response to light/dark transitions and low-temperature treatments. In a natural open-pollinated population, strong associations were observed between *CaUBC33/43* and the chlorophyll biosynthesis gene *CaCPOX*, as well as between *CaUBC33* and *Ca06g24560* (*r* = 0.7531, *p* < 0.0001) or *Ca00g64500* (*r* = 0.6287, *p* < 0.0001). These findings suggest that the protein ubiquitination pathway is involved in fruit pigment metabolism in pepper. This study provides critical insights into the functional roles of *CaUBC* genes in pigment biosynthesis and lays a solid foundation for further investigations into the molecular mechanisms regulating pigment homeostasis.

**Conclusions:**

The genome-wide analysis of UBC E2 ligases provides essential genomic resources for functional studies on *UBC* genes and is significant for the molecular breeding of pepper.

**Supplementary Information:**

The online version contains supplementary material available at 10.1186/s12864-026-12536-x.

## Background

The Ubiquitin-Proteasome System (UPS) is one of the most important pathways for specific protein degradation and constitutes an essential process contributing to normal plant growth [1]. The protein ubiquitination process is a multistep reaction that depends on the concerted action of three enzymes with catalytic activity, including ubiquitin-activating enzyme (E1), ubiquitin-conjugating enzyme (E2), and ubiquitin ligase (E3) [2]. Ubiquitin is conjugated to target proteins via a series of enzymatic reactions, and finally, the 26 S proteasome degrades the target protein. In this enzymatic cascade, the UBC enzyme is a central part for the attachment of ubiquitin to the degraded protein [3]. Most UBC enzymes have a highly conserved catalytic core, known as the UBC domain, which consists of approximately 150 amino acids (aa) and contains an active-site cysteine residue [4, 5]. Aside from the conserved UBC domain, some E2 enzymes have diverse amino acid terminal extensions that contribute to the intracellular localization or substrate specificity [6, 7]. Based on the conserved UBC domain and diverse N/C- terminal extensions, UBC enzymes can be divided into four classes: Class I UBCs contain only a typical UBC domain; Class II have an N-terminal extension and UBC domain; Class III harbor a UBC domain and C-terminal extension; and Class IV UBCs possess both the N- and C- terminal extensions as well as the UBC domain [8].

The E2 enzymes are reported to be widely involved in plant development and responses to biotic and abiotic stresses. *UBC* genes play crucial roles in flowering [9], seedling photomorphogenesis [10], ovule growth [11], root development [12], organ size regulation [13], fruit ripening [14], as well as in responses to various environmental stressors, such as drought [15–17], salt [18, 19], low temperature [20], and in sugar metabolism [21]. Genome-wide identifications of the UBC gene family have been carried out in many other plant species. Forty-eight UBC domain-containing proteins were identified in Arabidopsis [22]. In addition to 37 typical E2s, there are three thioester-linked UBLs, one SUMO-conjugating enzyme (AtSCE1), and two RUB conjugating enzymes (RCE1 and RCE2); these proteins perform E2-like functions but do not belong to the UBC family [22]. In addition, there are eight ubiquitin conjugating enzyme variants (UEVs) that lack the active-site cysteine residue [22]. To date, multiple UBC gene family members have been identified at the genome-wide level in several plant species, including sorghum (*Sorghum bicolor*) [23], potato (*Solanum tuberosum*) [24], papaya (*Carica papaya*) [25], rice (*Oryza sativa*) [26], tomato (*Solanum lycopersicum*) [27], upland cotton (*Gossypium hirsutum*) [17], wheat (*Triticum aestivum*) [28], grape (*Vitis vinifera*) [29], *Brassica napus* [30], strawberry(*Fragaria ananassa*) [31], and *Salvia castanea* [32]. However, systematic investigations of the UBCs in pepper is limited.

Pepper, a Solanaceae plant of great agricultural importance, has significant economic and nutritional value. Despite the advances in understanding the diverse and pivotal roles of *UBCs* in plant physiological and biochemical processes, only a few studies have been reported in pepper, especially functional studies of *UBC* genes. Existing research mainly focused on normal chromosome segregation [33], grain size and weight [13, 34], female gametophyte development [11], salt stress tolerance [19], and phosphorus homeostasis [35]. In this study, 56 *CaUBCs* were comprehensively identified, including their phylogenetic relationships, chromosomal locations, gene structures, conserved motifs, expression patterns in fruit at different developmental stages or under different temperature/light treatments, and co-expression with pigment biosynthesis genes or *U-box* E3 genes were investigated. Co-expression analysis uncovered that *CaUBC33/43* were significantly co-expressed with *U-box* E3 genes *Ca06g24560*, *Ca00g64500*, and the chlorophyll biosynthesis gene *CaCPOX*. The results of this study will help us to better understand the CaUBC enzyme and lay the foundation for future research on the functions of *CaUBCs* and their application in molecular breeding.

## Materials and methods

### Sequences data resources

A total of 48 Arabidopsis AtUBCs, including 37 typical AtE2 enzmyes, 8 UEVs, 1 SUMO-conjugating enzyme, and 2 RUB-conjugating enzymes, were downloaded from the TAIR database (www.arabidopsis.org). The pepper genome was queried using these AtUBC sequences as BLASTp queries on the Sol Genomics Network (http://solgenomics.net) with default parameters. The putative CaUBC proteins were submitted to the online tool SMART (http://smart.embl-heidelberg.de) for domain validation and those lacking UBC domains were excluded. The resulting 56 CaUBCs, including their corresponding genes, cDNAs, proteins and 2-kb promoter regions, were downloaded from the Sol Genomics Network. Additionally, their physicochemical and genetic properties, such as genomic location, protein length, gene structure, protein molecular weight, and isoelectric point (pI) were also obtained. Cis-acting element analysis was performed by the PlantCARE tool (http://bioinformatics.psb.ugent.be/webtools/plantcare/html). The chromosomal positions of *CaUBC* genes were visualized using MapInspect software.

### Phylogenetic and motif analysis

All UBC proteins, including 48 AtUBCs, 52 SlUBCs, and 56 CaUBCs, were aligned using the online MAFFT tool with default settings (http://mafft.cbrc.jp). After deleting unconserved regions, and the conserved sequences were subsequently used to construct a phylogenetic tree by IQ-TREE with 1000 bootstrap replicates, while other parameters were set to default (http://iqtree.cibiv.univie.ac.at/). The MEME program was used to search for the conserved motifs in CaUBC proteins with default settings (http://meme-suite.org). TBtools (version 2.102) was used to illustrate the results [36].

### Plant materials and treatment

A natural open-pollinated segregating population (NOS) derived from the genetic background of Da-Fang wrinkled pepper and Bi-Jie line pepper was used in this study. Both research materials were provided by the Chili Pepper Research Institute, Guizhou Academy of Agricultural Sciences.

The seeds were surface-sterilized using a 2% sodium hypochlorite solution, rinsed thoroughly with sterile water, and germinated in Petri plates. The seedlings were then transferred to a nutrient solution (half-strength modified Hoagland’s solution), which was changed every 7 days. For light/dark or temperature treatments, uniform-sized flowering seedlings were transplanted into plastic pots and cultivated in a growth chamber under a 16 h/8 h light/dark photoperiod at 28 ℃. At the green mature fruit stage (approximately 40 days post-anthesis, DPA), seedlings were subjected to dark conditions or 20 ℃ treatment at 0 h, 1 h, and 12 h. The pericarp and placenta were sampled, immediately immersed in liquid nitrogen, and stored at −80 ℃. To detect the expression profiles of *CaUBCs* during fruit development, seedlings were planted in plastic pots and grown at Qingzhen, Guiyang. At the time of fruiting, fruit samples were collected at 10, 20, 30, 40 and 50 DPA, rapidly frozen in liquid nitrogen, and preserved at – 80 ℃. For co-expression analysis, seedlings from NOS population with Da-Fang wrinkled line pepper genetic background were grown at Guizhou Normal university. Fruits at the green mature stage were collected, rapidly frozen in liquid nitrogen, and stored at −80 ℃ for subsequent use.

### RNA isolation and qRT-PCR

Total RNA from the pericarp and placenta of pepper fruits was isolated using RNAiso Plus (Takara Biotechnology, Dalian, China) according to the manufacturer’s instructions. First-strand cDNA was synthesized by reverse transcription using M-MLV reverse transcriptase (Takara Biotechnology, Dalian, China). Quantitative real-time PCR (qRT-PCR) was performed on a qTOWER thermal cycler (Analytic Jena Gmbh, Jena, Germany) with Hieff UNICON Universal Blue qPCR SYBR Green Master Mix (Yeasen, China). The pepper β-*ACTIN* gene was used as the internal control. Primers were designed using Oligo 7, and their sequences are listed in Supplementary Table 1. The PCR reaction system had a total of 10 µl, consisting of 5 µl of 2× SYBR Green reaction mix, 1 µl of cDNA template, 0.5 µl each of forward and reverse primers, and 3 µl of ddH_2_O. The PCR amplification procedure was as follows: 95 ℃ for 10 s, 55 ℃ for 20 s, and 72 ℃ for 20 s, with 40 cycles performed. Fluorescence signals were collected during the 72 ℃ extension step. Relative gene expression levels were calculated using the 2^−△△Ct^ method [37]. Statistical comparison of expression levels were performed using one-way analysis of variance (ANOVA) with a significance threshold of *p* < 0.05 in GraphPad Prism 8.0 software.

### Co-expression analysis in a natural open-pollinated population of pepper

A natural open-pollinated segregating population of pepper, comprising approximately 120 seedlings with rich fruit color variation phenotypes, was used to perform co-expression analysis between *CaUBCs* and either pigment biosynthesis genes or *CaU-box* genes. GraphPad Prism 8.0 was applied to perform association analysis based on the segregating population with the nonparametric Spearman correlation method.

## Results

### Screening and Identification of *UBC* genes in pepper

**Fig. 1:**
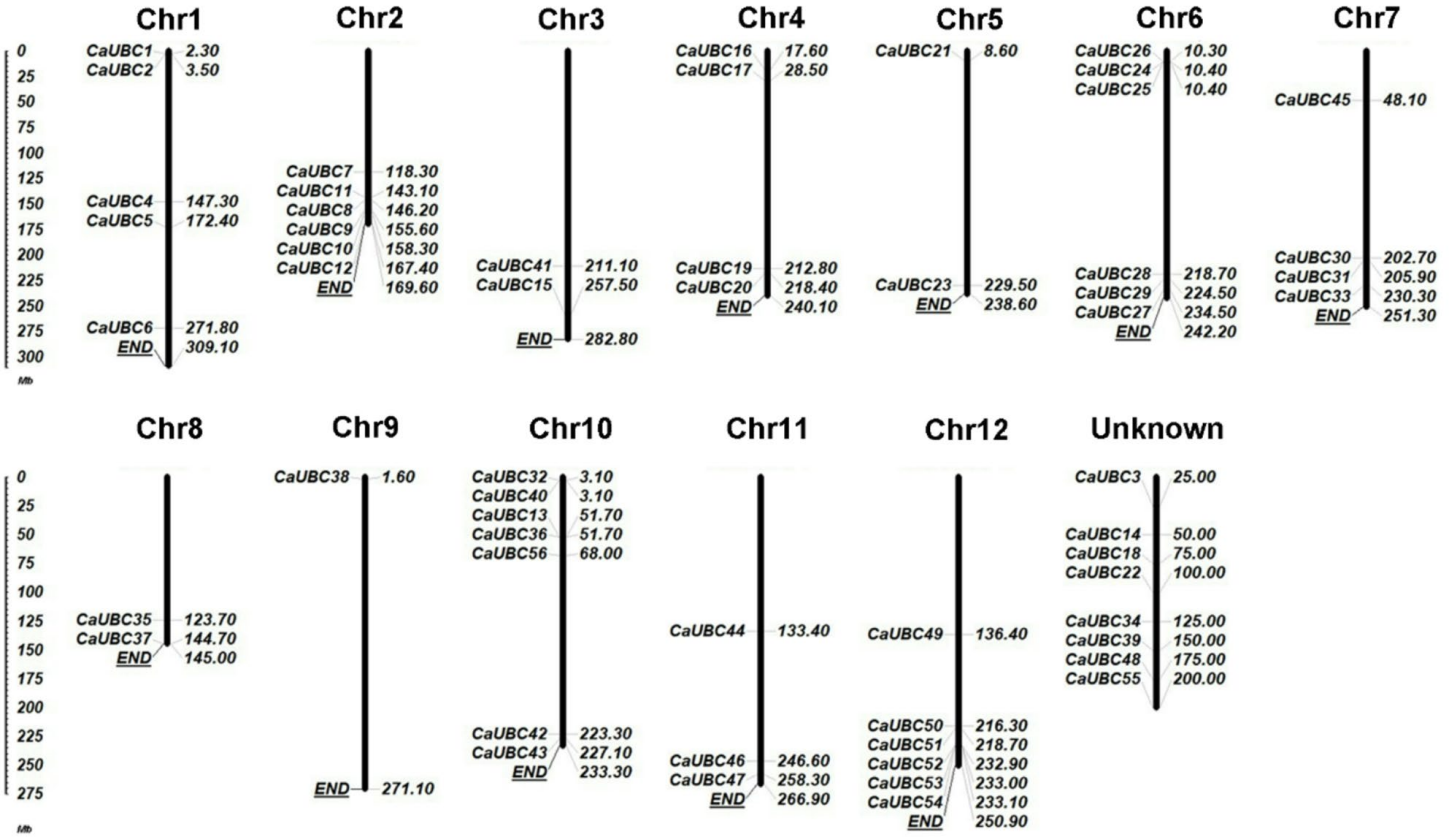
Chromosome localization analysis of the *CaUBCs* gene family in pepper. The scale bar on the left indicates chromosome length (Mb). Chromosome numbers are shown at the top of the bars. Genes are positioned on the right side, with their physical locations on the left side. The “Unknown” bar contains genes whose physical locations remain undetermined

After performing BLASTp sequence alignment, redundancy removal, and protein domain confirmation, 56 *CaUBC* genes were identified. These genes are unevenly distributed across 12 pepper chromosomes (Fig. 1): 6 members are present on chromosomes 1, 2, and 6; 4 members are found on chromosomes 7, 8, 10, and 11, respectively. The names and protein parameters of the 56 *CaUBCs* are shown in Table S2. The protein parameters of these 56 CaUBCs varied significantly: for instance, their protein lengths range from 89 aa (CaUBC4) to 1154 aa (CaUBC37), with relative molecular weights ranging from 9.98 kDa to 127.56 kDa. Additionally, the overall isoelectric point (pI) values range from 4.47 to 9.70. Gene structure analysis revealed considerable variation in the number of exons and introns within the *CaUBC* gene family (Additional file 1 Figure S1.). *CaUBC4*,* 5*,* 24*,* 26*,* 29*,* 30*,* 38*,* 45*,* 47*,* 48*,* 53*, and *56* contain fewer than two introns, while *CaUBC1*,* 2*,* 3*,* 6*,* 7*,* 9*,* 10*,* 11*,* 12*,* 13*,* 14*,* 16*,* 17*,* 18*,* 19*,* 22*,* 25*,* 28*,* 33*,* 34*,* 35*,* 36*,* 41*,* 42*,* 43*,* 44*,* 46*,* 49*,* 50*,* 51*, and *54* have three to four introns. The remaining members typically have 5–8 introns, with *CaUBC55* having the highest number (9 introns). Conserved gene structures were observed within the same group; for example, each *CaUBC* in group IV contains 3 or 4 conserved introns.

### Phylogenetic and motif analysis

**Fig. 2:**
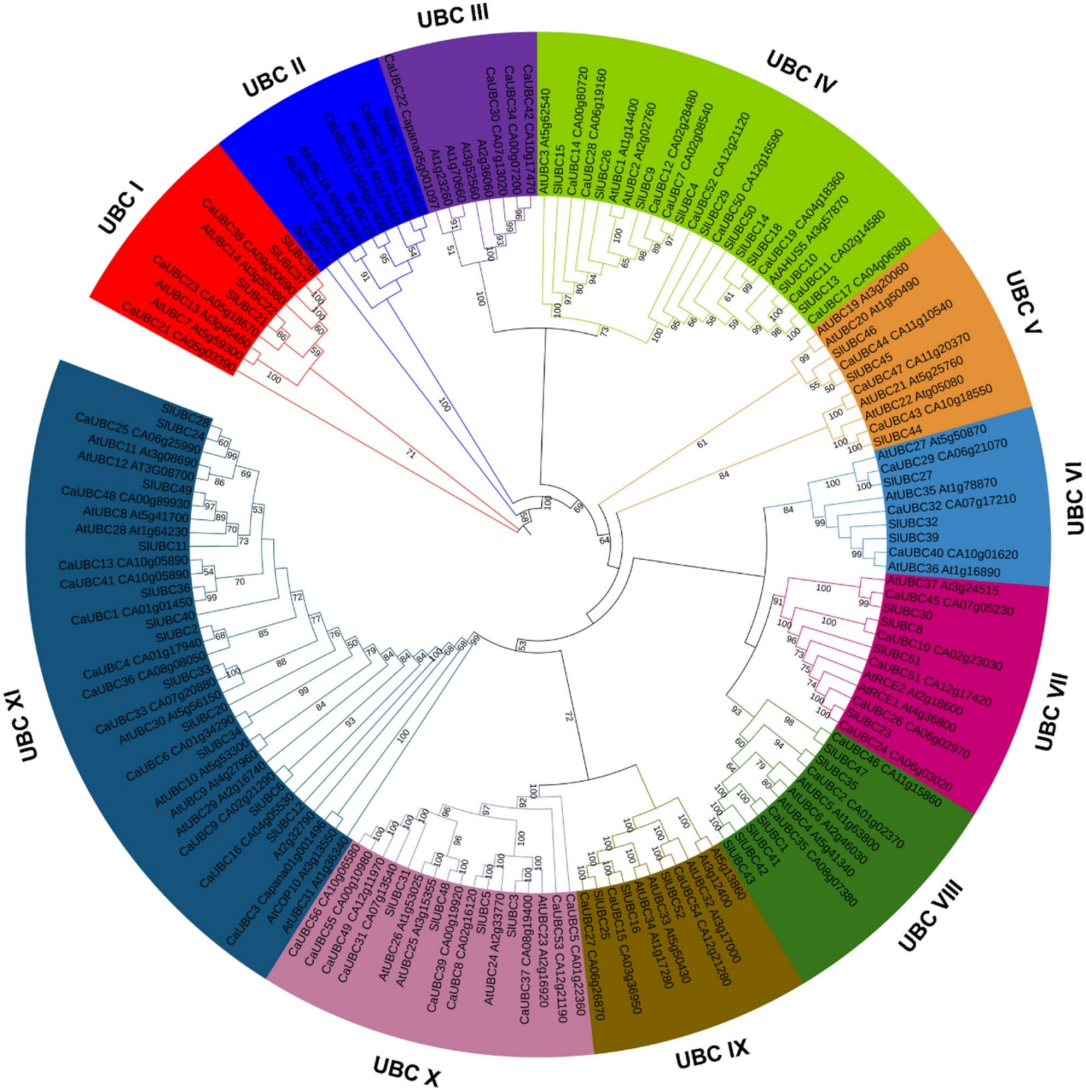
Phylogenetic analysis of CaUBC proteins. Different colors represent various subfamilies of the UBC gene family. The UBC gene family comprises 11 subgroups, each designated by its respective name

To better understand the evolutionary relationships among *UBC* genes, phylogenetic trees were constructed, and comparisons of gene structures and protein conserved motifs were conducted. A total of 155 UBC proteins, including 56 CaUBCs, 48 AtUBCs, and 52 SlUBCs, were used to construct a phylogenetic tree by IQ-TREE online tool. The results showed that these protein sequences could be divided into 11 groups, namely UBC I to UBC XI, including both typical and atypical UBC clades. For example, group UBC VII contains UEVs, group UBC IV contains HUS proteins, and group UBC XI contains COP10 (Fig. 2). An unequal distribution of gene numbers per clade was observed. For example, group UBC XI has 12 *CaUBC* genes, while groups UBC I, UBC II, UBC III, UBC IV, UBC VIII and UBC IX each contain only 2 to 3 *CaUBCs*. Notably, the UBC III group lacks tomato homologs, probably due to gene loss during evolution. *UBC* genes from all three species are present in almost every group, indicating that the evolutionary history of the *CaUBC* gene family is consistent with that of tomato and Arabidopsis. Similar results have been reported in other plants [31, 32].

**Fig. 3:**
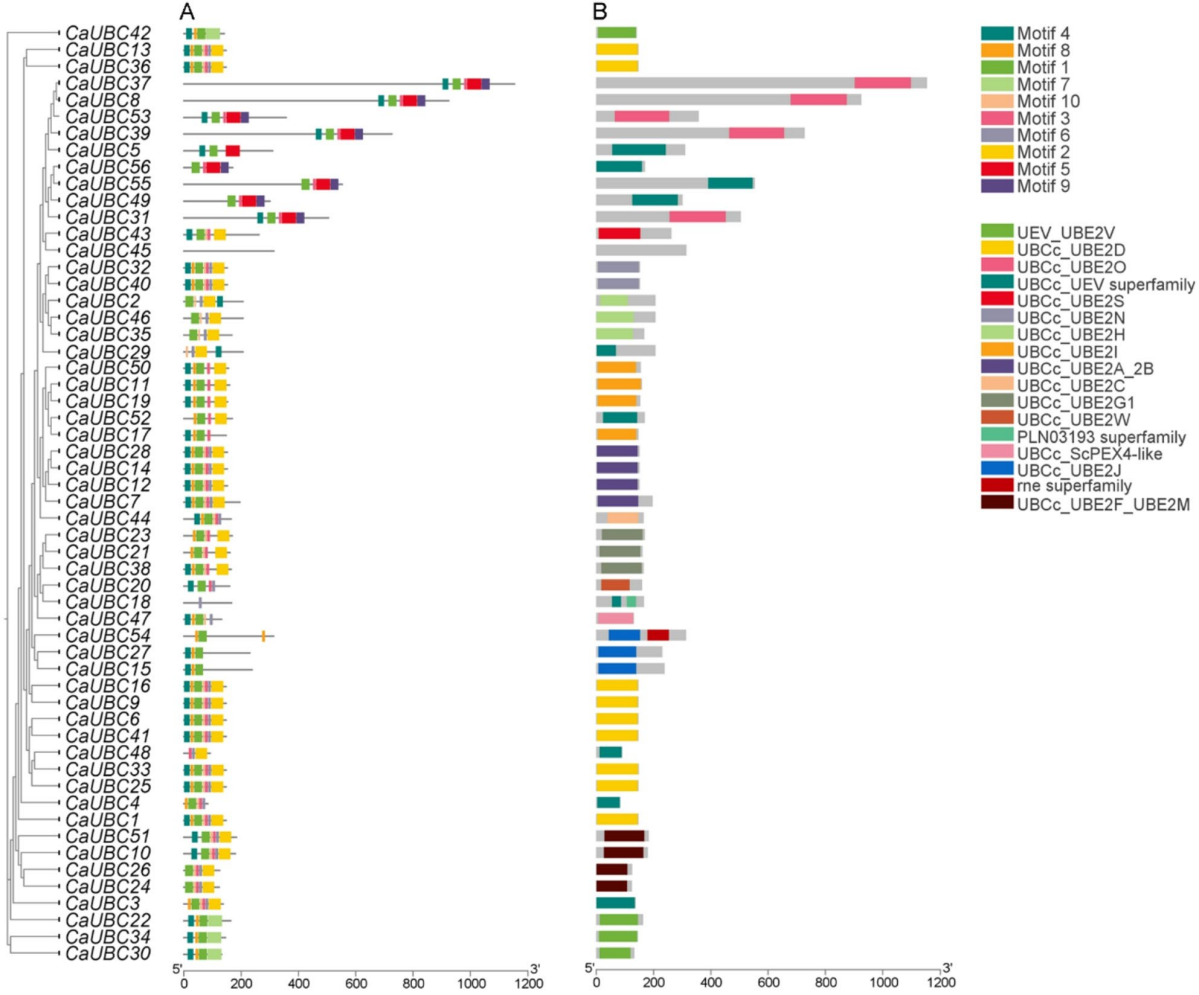
Conserved motif and domain distribution of CaUBCs. The colored boxes denote various conserved motifs and domains. **A** Schematic presentation of the conserved motifs of CaUBC proteins; (**B**) Schematic presentation of the domains of CaUBC proteins

The conserved motif analysis of 56 CaUBC proteins was performed by the MEME online tool (Fig. 3A). Ten motifs were identified, with amino acid sequence lengths ranging from 8 to 50 aa. All CaUBC proteins, except CaUBC45, contained at least one motif. Motif 1, a highly conserved motif, was present in all protein sequences except those of CaUBC18 and CaUBC48. Additionally, motif types were conserved within each phylogenetic group; for example, group XI consisted of 12 CaUBC proteins, all of which share similar motif types (motifs 1, 2, 3, 4, 6, 8, and 10), with the exception of CaUBC48. Different motifs were composed of distinct domains and showed variations among different phylogenetic groups (Fig. 3B).

### Cis-acting element analysis

**Fig. 4:**
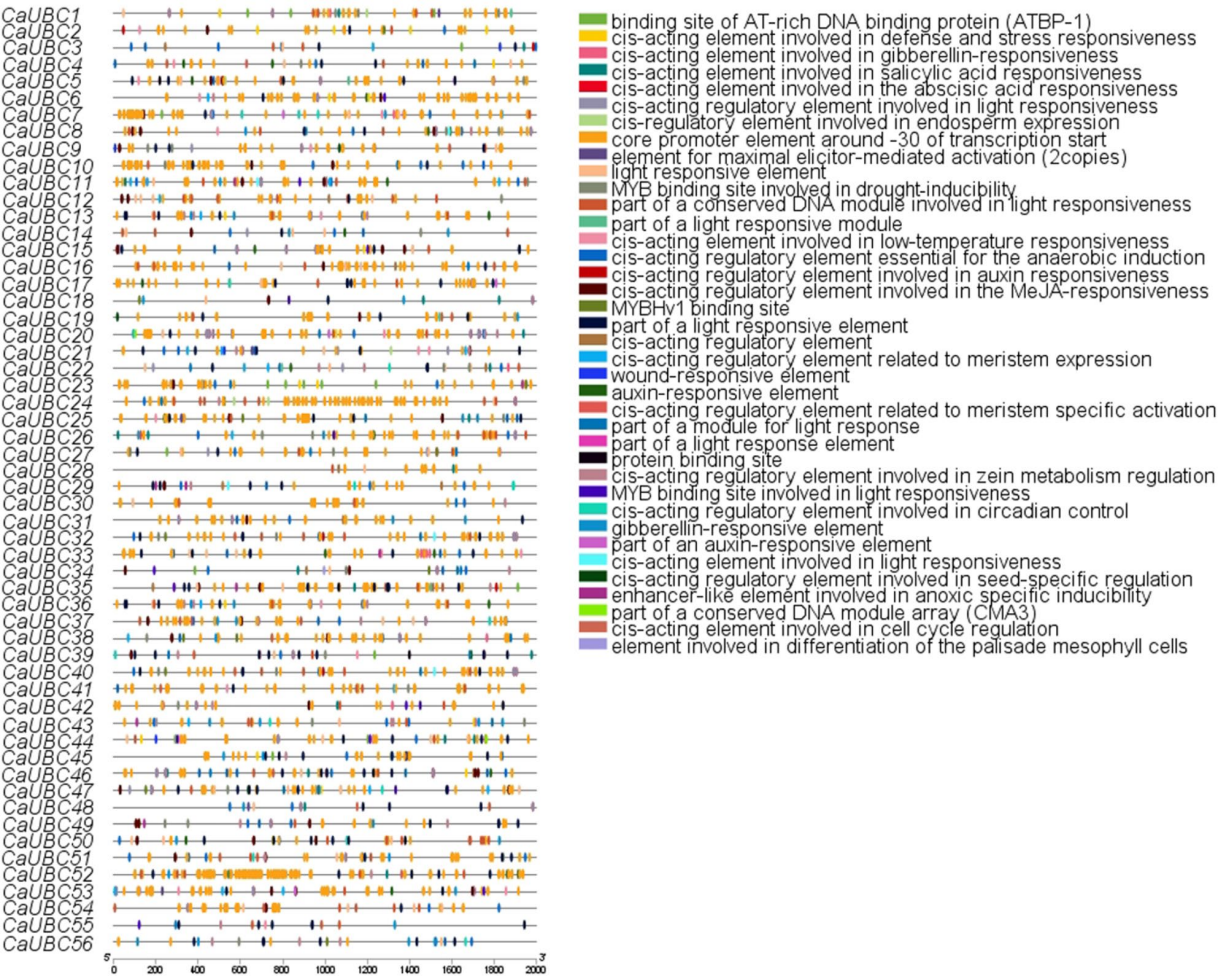
Cis-acting elements of *CaUBC* genes in pepper. The distribution of cis-acting elements in the promoters of 56 *CaUBC* genes is illustrated. Colored boxes on the black lines represent different cis-acting elements. Cis-acting elements were predicted using the online tool PlantCARE and visualized with TBtools II

To explore the potential functions of *CaUBC* genes, cis-acting elements were predicted using the PlantCare tool, and multiple cis-acting elements were identified, including light responsive elements, hormone responsive elements, stress responsive elements, and plant growth related elements (Fig. 4). *CaUBCs* have the largest number of light-responsive elements, with a total of 520 identified, including typical ones such as G-box, GT1-motif, MRE, box4, ATCT motif, I-box. Stress-responsive elements in CaUBCs rank second in number, predominately those induced by cold and drought. These findings suggest that *CaUBC* genes may be regulated by various environmental factors (e.g., light, cold, drought) and could be involved in plant growth and development mediated by these factors.

### Expression analysis of the *CaUBC* genes in pepper fruit development

**Fig. 5:**
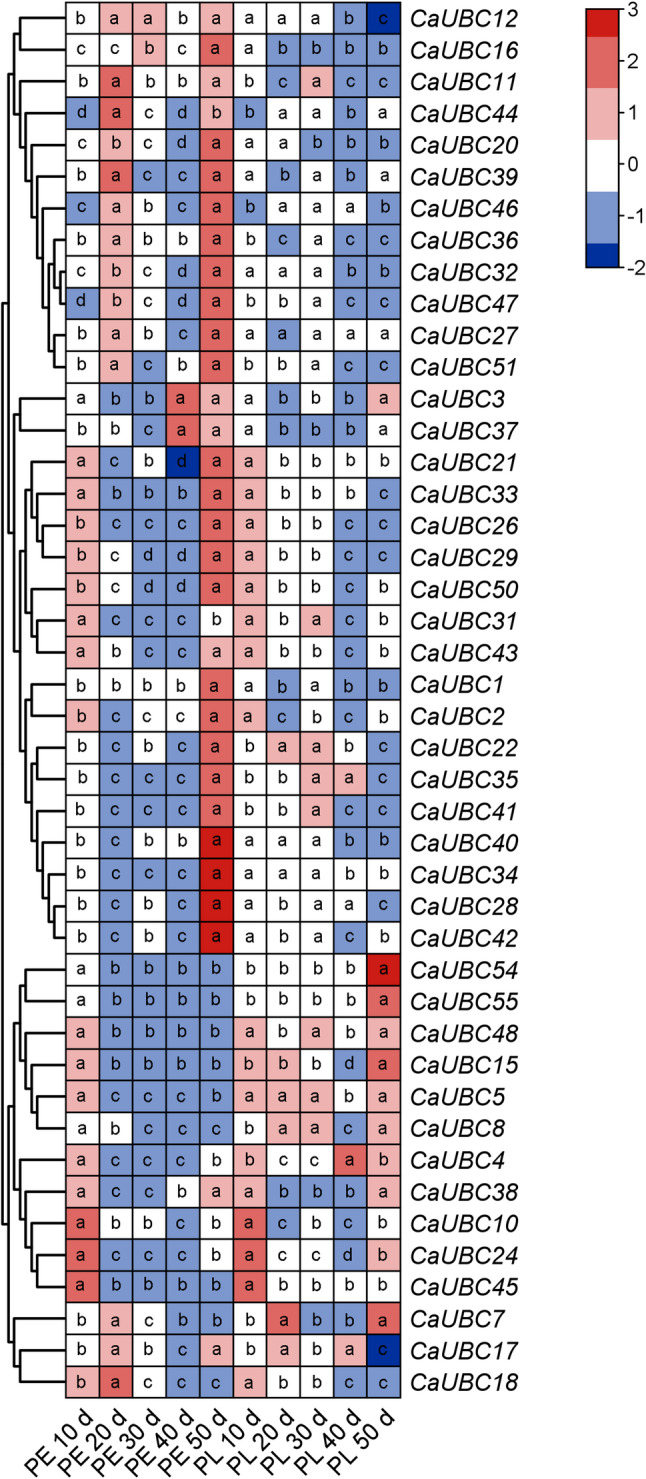
Expression profiles of *CaUBC* genes in pepper fruit at different developmental stages are shown by the heatmap. PE, pericarp; PL, placenta, Blue represents low expression, whereas red indicates high expression. Lowercase letters in the boxes indicate significant differences (α = 0.05, LSD) among the treatments

To understand the comprehensive expression profiles of all *CaUBC* genes during pepper fruit development, the Bi-Jie line pepper was employed as the research material. qRT-PCR was utilized to detect the expression levels of *CaUBCs* during fruit development (Fig. 5). The results showed that *CaUBCs* exhibited diverse expression patterns in the pericarp and placenta. The expression levels of *CaUBC10*,* 18*,* 24*,* 31*,* 38*,* 43*, and *45* decreased significantly in both tissues; for instance, *CaUBC18* and *CaUBC43* were downregulated by at least 85% compared to the control at 40 DPA, suggesting these genes may exert similar functions during the fruit ripening. Only *CaUBC11* exhibited consistent upregulation throughout fruit development. The expression patterns of other genes were somewhat diverse. For example, the expression levels of *CaUBC7* were upregulated by at least 5-fold in both tissues before 20 DPA, but then decreased significantly at 40 DPA. *CaUBC16* was consistently upregulated in the pericarp, especially by > 50-fold at 50 DPA, while being obviously downregulated in the placenta, indicating its opposite functional roles in pericarp versus placental development. Several genes, such as *CaUBC20*,* 27*,* 28*,* 32*,* 36*,* 39*,* 40*,* 42*,* 44*,* 45*,* 46*, and *51*, were upregulated by more than 10-fold in the pericarp at 50 DPA but not in the placenta, implying tissue-specific functional roles at this stage.

### Expression pattern under different light intensity and temperature

**Fig. 6:**
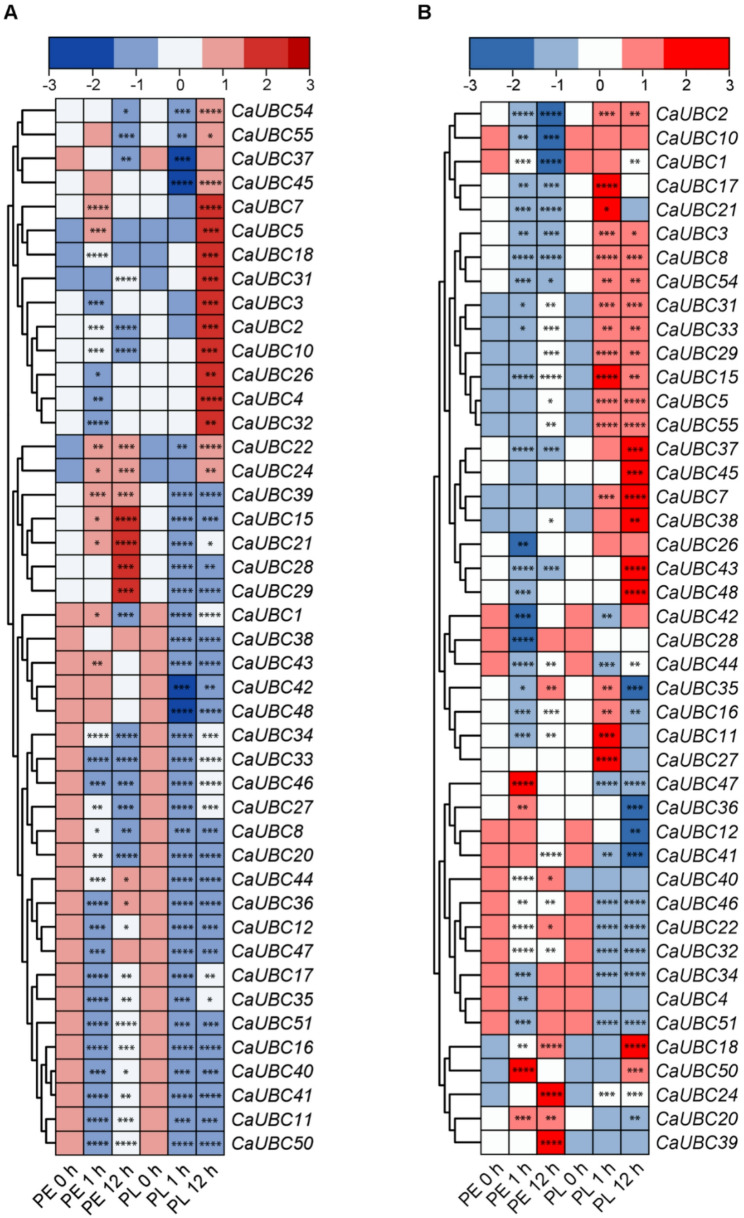
Expression patterns of 56 *CaUBC* genes in response to light/dark (**A**) or 28/20 ℃(**B**) transition at 0 h, 1 h, and 12 h are illustrated by the heatmap. Blue represents low expression, whereas red indicates high expression. ༊p-value < 0.05; ༊༊p-value < 0.01; ༊༊༊p-value < 0.001; ༊༊༊༊p-value < 0.0001

Fruits are a vital source of essential vitamins and a diverse array of bioactive compounds, including carotenoids, polyphenols, and polyunsaturated fatty acids. Fruit ripening is a complex process regulated by both internal and external factors, such as various regulatory genes, light, and temperature. To investigate the potential functions of *UBC* genes in these biological processes, the expression levels of *CaUBCs* in the pericarp or placenta were detected using qRT-PCR under light/dark transitions or 28℃/20℃ temperature shifts at 0 h, 1 h, and 12 h (Fig. 6A, B). The results showed that most *CaUBC* genes in the pericarp or placenta were downregulated under both treatments. For example, *CaUBC22*,* 24*,* 32*,* 34*,* 41*,* 44*, and *46* were significantly downregulated in response to light/dark transitions, while *CaUBC1*,* 4*,* 12*,* 22*,* 28*,* 32*,* 34*,* 41*,* 44*, and *46* had obviously decreased expression at 20 ℃. Only *CaUBC31* and *CaUBC24* in the pericarp and placenta were simultaneously upregulated under both treatments. These findings indicate that light or high temperature had a negative regulatory effect on *CaUBC* expression. The result is similar to a previous report that light plays a negative regulatory roles in regulating the *MAPK* gene family [38].

In addition, the expression patterns of certain *CaUBC* genes showed opposite trends in the pericarp versus placenta. For example, under dark conditions, *CaUBC15*,* 21*, and *39* were upregulated in the pericarp but obviously downregulated in the placenta, whereas *CaUBC3*,* 8*,* 20*,* 37*, and *54* were significantly downregulated in the pericarp while being upregulated in the placenta. These results indicated that *CaUBC* genes may play contrasting functions in the pericarp and placenta, highlighting the complex roles of the *CaUBC* gene family during pepper fruit development.

Co-expression analysis between *CaUBC* and pigment biosynthesis gene

**Fig. 7:**
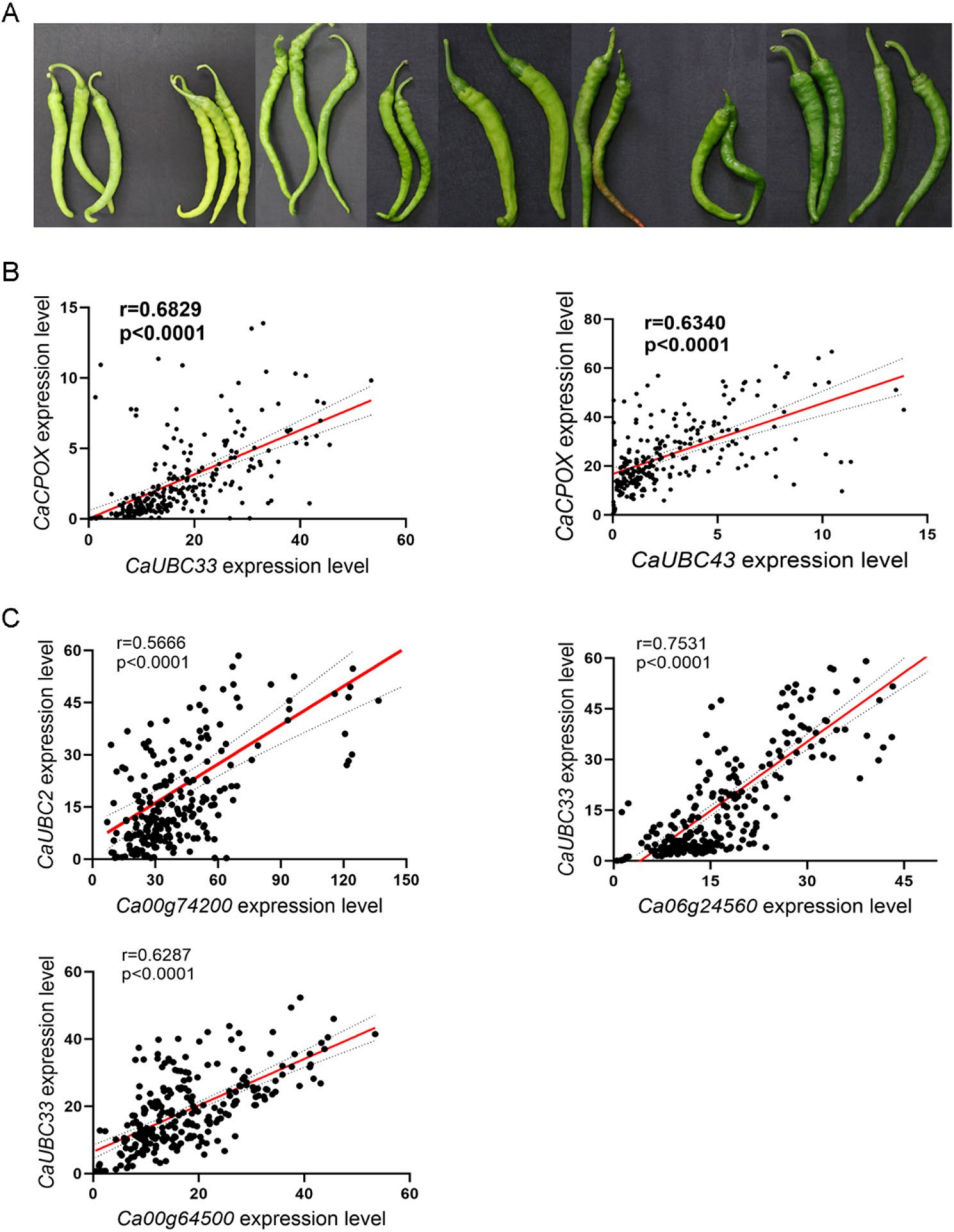
Correlation analysis between *CaUBCs* and pigment biosynthesis gene *CaCPOX* or *CaU-box* genes. **A** Fruit color variation in the NOS population. From left to right, the fruit color gradually darkens, ranging from light green to dark green. **B** Co-expression analysis between *CaUBC* and pigment biosynthesis gene *CaCPOX*. **C** Correlation analysis between *CaUBC* and *CaU-box* genes

Ubiquitination is an important post-translational modification that mediates target protein degradation and participates in multiple biological processes during plant development. In recent years, some studies have reported that ubiquitination-mediated proteolysis is involved in fruit pigment homeostasis [40–45]. To explore the relationship between the *CaUBC* gene family and pigment biosynthesis, we scrutinized the expression levels of both *CaUBC* family genes and pigment biosynthesis genes using a NOS population in Da-Fang wrinkled line pepper genetic background (Fig. 7, Additional file 2 Figure S2). This population has plenty of different fruit color phenotypes (Fig. 7A). The results revealed that *CaUBC33* and *CaUBC43* share similar expression patterns with the chlorophyll biosynthesis gene *CaCPOX*, and these trends were not driven by occasional several individuals. To validate these observations, the segregating population was expanded, and a nonparametric Spearman correlation analysis was employed to quantify the association strength between *CaUBC33/43* and *CaCPOX* based on relative expression values. The results showed that *CaUBC33/43* were most strongly associated with *CaCPOX* (*CaUBC33/CaCPOX*, *r* = 0.6829, *p* < 0.001, *CaUBC43/CaCPOX*, *r* = 0.6340, *p* < 0.0001, Fig. 7B), indicating that members of the *CaUBC* family are involved in chlorophyll biosynthesis. A previous report showed that the tomato regulator RIN can directly bind to the E2 promoter region, forming a regulatory module that controls tomato fruit color [14]. Future work should focus on the exact biological functions of *CaUBCs* in pigment homeostasis.

U-box E3 ligase usually binds to E2 enzymes by the U-box domain to target proteins for degradation. We conducted a co-expression analysis between *CaUBCs* and *CaU-box* genes in mature green fruits from the NOS population (Fig. 7C, Additional file 2 Figure S2). The results showed that *CaUBC2* has a moderate correlation with *Ca06g24560* (*r* = 0.4525, *p* < 0.0001), *Ca00g64500* (*r* = 0.4561, *p* < 0.0001), and *Ca00g74200* (*r* = 0.5666, *p* < 0.0001); *CaUBC33* has a strong correlation with *Ca06g24560* (*r* = 0.7531, *p* < 0.0001), and *Ca00g64500* (*r* = 0.6287, *p* < 0.0001). the results suggest that these protein pairs might form protein complexes to regulate fruit color formation in pepper.

## Discussion

Gene duplication events have a higher percentage in plant genomes than in those of other eukaryotes [45]. With the development of plant genome project, expansion and contraction of gene families have been widely reported across various plant species [46]. As central components of the protein ubiquitination process, E2 enzymes have been identified in multiple plant species, with the number of UBCs varying significantly among them. In this study, a total of 56 *CaUBCs* were identified, a number similar to that of the congeneric species tomato (59 *SlUBCs*) [27]. This count is significantly lower than in strawberry [31], wheat [28], banana [47], or maize [48], but higher than in Arabidopsis [22] and rice [26]. Given that the genome sizes of pepper, tomato, strawberry, wheat, banana, maize, Arabidopsis, and rice are approximately 3080, 900, 523, 16,000, 900, 2300, 125, and 466 Mb, respectively, it is evident that the number of UBC enzymes does not correlate with genome size in a simple linear manner. This indicated that gene family expansion or contraction is not solely driven by the whole genome duplication; segmental or tandem duplication also plays important roles.

Despite the significance of UBC family members during plant growth, only a few studies have been reported in horticultural plants, such as tomato [14], banana [48], papaya [25], and *Vitis vinifera* (grape) [29]. Tomato *UBC32/41* is involved in fruit ripening and is regulated by tomato ripening regulator *RIN* gene [14]. In grape, most *VvUBCs* in five Italian varieties exhibit expression changes during ripening, with their levels gradually decreasing at the softening stage [29]. this trend partially aligns with that of *CaUBC* genes: most *CaUBCs* gradually declined at the green mature stage and then obviously increased. Similar results have been reported in tomato [14]. However, opposing expression trends were observed in *SlUBC1*,* 3*,* 4*,* 6*,* 16*,* 18*,* 37*,* 40*, and *45*, which show higher expression at the green mature stage followed by gradual declined until red ripe stage [14]. Their corresponding closest pepper homologs *CaUBC35*,* 37*,* 7*,* 9*,* 15*,* 19*,* 38*,* 41*, and *44* exhibit diverse expression patterns. The distinct expression differences among horticultural fruits indicate species-specific functional differentiation of UBC during fruit development. Differential UBC gene expression is also exhibited in strawberry: *FaUBC* genes were classified into 6 clusters based on their expression patterns, with cluster 2 showing gradually increasing expression during fruit development, while cluster 3 exhibited a sudden upregulation at the TR stage [31]. Species-specific expression patterns are further distinct in response to temperature changes. Previous reports showed that E2 genes in both *Arabidopsis* and rice are not significantly altered under cold stress [49], while maize *UBCs* changed significantly [48]. In grape, seven *VaUBCs* responded to cold treatment while six *VaUBCs* are markedly altered under heat conditions [29]. Their closest homologous *CaUBC* genes display varied expression patterns in the pericarp and placenta, supporting species-specific differentiation in expression.

Pepper, a member of the large Solanaceae family, is one of the most extensively cultivated vegetable crops globally. Fruit color, a critical indicator of pepper fruit quality, has made some progress in research on its genetic and molecular regulatory mechanisms. Variations in fruit color arise from the relative abundance of pigments such as chlorophyll, anthocyanins, carotenoids, and other pigments, resulting in extensive diversity. Significant progress has also been made in understanding the steps of pigment biosynthesis, including those of chlorophyll, anthocyanins, and carotenoids. Several key regulators have been identified in the processes of pigment biosynthesis and degradation. A number of transcription factors are known to regulate chlorophyll synthesis, including LOL1 [50], MdBEL7 [39], MdERF17 [38] and MdEIL [51], and, GLK2 [40]. Additionally, CrWRKY42 [52], R2R3-MYB [53], ERF5.1 [54], CaMYB31 [55] and CaMYB48 [56], FvRIF [57], FaMYB10 [58], FvDFR2 [59] and FvPAL2 [60] are involved in carotenoid biosynthesis, while VviNAC60 [61] and MYB24 [62] participate in anthocyanin biosynthesis. Notably, the ubiquitin-26 S proteasome pathway also plays an important role in regulating pigment biosynthesis. For instance, tomato E2 genes (*SlUBC6*,* 8*,* 24*,* 32*,* 41*, and *42*) are directly regulated by the fruit-ripening regulator RIN; silencing of *SlUBC32* or *SlUBC41* leads to abnormal fruit pigmentation [14]. Moreover, these genes show differential expression between wild type and RIN mutant fruits, with peak expression at orange stage. The closest pepper homologs of *SlUBC32* and *SlUBC41*, *CaUBC32* and *CaUBC35*, exhibit similar expression patterns, suggesting conserved roles of these homologous pairs in pigment regulation across tomato and pepper. In this study, using NOS population with rich fruit color variation, a strong association was observed between *CaUBC33*,* CaUBC43* and chlorophyll biosynthesis gene *CaCPOX*. The significant downregulation of these two *CaUBC* genes during fruit development indicates they may play negative regulatory roles in fruit color formation. This is further supported by findings in strawberry, where *FaUBC76/78* have distinct roles in anthocyanin biosynthesis [31]; notably, *CaUBC33* is the closest homolog of FaUBC76, reinforcing its potential function in regulating chlorophyll biosynthesis in pepper. The exact molecular functions and regulatory mechanisms of these genes will be explored in subsequent studies.

The biosynthesis and metabolism of pigments involve a complex dynamic equilibrium, which is coordinately regulated by genetic and environmental factors [63]. In recent years, numerous studies have reported that the protein ubiquitin degradation system is involved in regulating pigment metabolic balance in some plants [64]. Our research provides comprehensive information on *CaUBCs*, and by utilizing the NOS population with segregating fruit colors, we identified *CaUBC33* and *CaUBC41* as genes co-expressed with *CaCPOX*, a key enzyme in the chlorophyll biosynthesis pathway. Additionally, we further explored *CaU-box* genes that may interact with these two genes. Based on the available data, it can be hypothesized that *CaUBC33* or *CaUBC41* interacts with CaU-box E3 ubiquitin ligases (*Ca06g24560* and *Ca00g64500*) to coordinately regulate the enzymatic activity or transcriptional activity of *CaCPOX*, thereby modulating fruit color. However, these results are mainly derived from bioinformatics analyses and co-expression association studies. Subsequent experiments will require diverse functional assays, such as functional validation and protein-protein interaction assays, to elucidate the functional roles of these genes.

## Conclusions

This study identified and systematically characterized 56 *UBC* genes in pepper, including chromosomal localization, cis-acting elements, conserved motifs and domains, phylogenetic relationships, qRT-PCR expression profiles, co-expression analysis, and association with potential interacting proteins. A total of 56 *CaUBC* genes were identified, and they are scattered across 12 chromosomes. The phylogenetic tree showed that CaUBC proteins can be classified into 11 groups, each containing conserved motifs, gene structures, or domains. Meanwhile, light responsive elements, stress related elements, and hormone response elements are abundantly present in the promoter regions of *CaUBCs*. Variable expression patterns of *CaUBCs* were observed during fruit development and in response to light/dark transitions, or 28/20 ℃ temperature shifts in pepper, suggesting that *CaUBCs* play versatile and important roles during fruit development and in response to various abiotic stresses. Association analysis using NOS population showed that the expression levels of *CaUBC33* and *CaUBC43* were strongly associated with the chlorophyll biosynthesis gene, *CaCPOX*, respectively. Additionally, *CaUBC33* was significantly associated with *Ca06g24560* (*r* = 0.7531, *p* < 0.0001), and *Ca00g64500* (*r* = 0.6287, *p* < 0.0001). These results indicate that protein ubiquitination pathway is involved in fruit color formation in pepper. The findings of this work lay a foundation for further functional analysis of the roles of *CaUBC* genes in pepper.

## Supplementary Information

Supplementary Material 1.

Supplementary Material 2.

Supplementary Material 3.

Supplementary Material 4.

## Data Availability

The AtUBC sequences were obtained from the Arabidopsis Information Resource (TAIR) database (https://www.arabidopsis.org). The SlUBC and CaUBC sequences were downloaded from the Sol Genomics Network (http://solgenomics.net). The datasets analysed during this study are included in this published article and its supplementary information files.
