# Supplementary material for: Characterization and expression analysis of UBC gene family provide insights into the potential roles in pigment biosynthesis in pepper fruit
Source: BMC Genomics. 2026 Jun 9;27:529. doi: 10.1186/s12864-026-12536-x (PMC13248304; doi:10.1186/s12864-026-12536-x)
Supplement: Supplementary file 1 — Supplementary Material 1. [file 12864_2026_12536_MOESM1_ESM.docx]

Supplementary Information


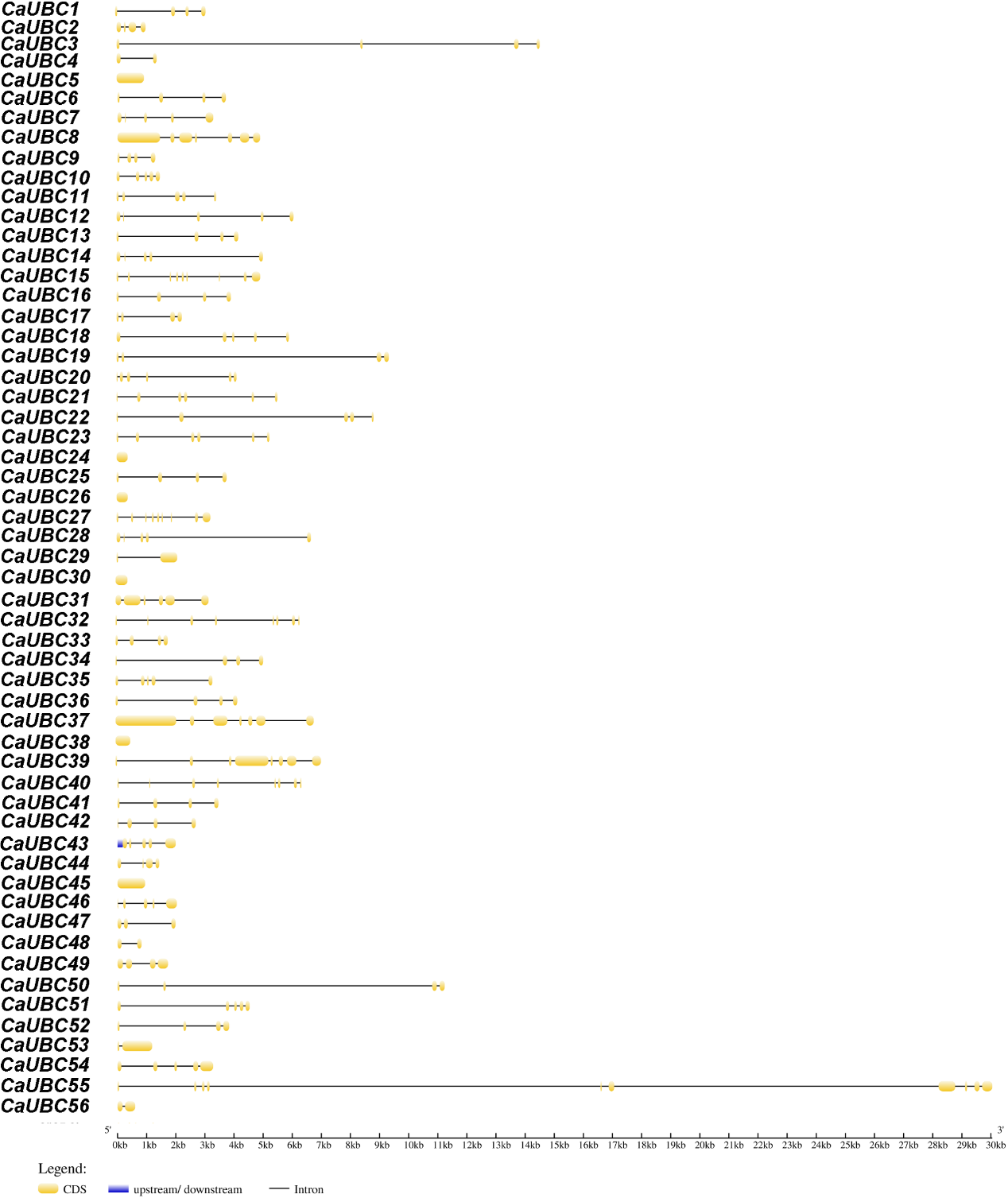


Figure S1. Gene structure of 56 *CaUBC* genes was analyzed using the GSDS tool.
